# A Comparative Study on Na_2_Fe_0.6_Mn_0.4_PO_4_F/C Cathode Materials Synthesized With Various Carbon Sources for Na-ion Batteries

**DOI:** 10.3389/fchem.2020.633949

**Published:** 2021-01-13

**Authors:** Shibao Tang, Xiaoping Zhang, Yulei Sui, Bingjue Wang, Jiangpeng Li, Ling Wu

**Affiliations:** School of Iron and Steel, Soochow University, Suzhou, China

**Keywords:** sodium-ion batteries, cathode materials, Na_2_Fe_0.6_Mn_0.4_PO_4_F, spray drying, electrochemical performance

## Abstract

Na_2_Fe_0._6Mn_0.4_PO_4_F/C composite materials are synthesized with various carbon sources via a simple spray-drying method in this study, and the effect of carbon sources on structure, morphology, and electrochemical properties of Na_2_Fe_0.6_Mn_0.4_PO_4_F/C materials are investigated in detail. XRD and SEM results indicate that the reduction ability of carbon sources has a key impact on the structure and morphology of Na_2_Fe_0.6_Mn_0.4_PO_4_F/C composite materials. Among these Na_2_Fe_0.6_Mn_0.4_PO_4_F/C materials, the sample prepared with ascorbic acid presents a uniform hollow spherical architecture. Electrochemical analysis demonstrates that the Na_2_Fe_0.6_Mn_0.4_PO_4_F/C sample prepared with ascorbic acid has optimal electrochemical performance. The sample shows high discharge capacities of 95.1 and 48.1 mAh g^−1^ at 0.05C and 1C rates, respectively, and it exhibits an improved cycle stability (91.7% retention after 100 cycles at 0.5C), which are superior to Na_2_Fe_0.6_Mn_0.4_PO_4_F/C materials prepared with other carbon sources. This study demonstrates that the reduction ability of carbon sources significantly influences the electrochemical properties of fluorophosphate/C composite materials. This work also provides a promising strategy to obtain high performance cathode materials for sodium-ion batteries.

## Introduction

In recent years, the demand for lithium-ion batteries (LIBs) has increased sharply due to the rapid development of large-scale energy storage and electric vehicles (Wu et al., 2016; Li et al., 2020; Nie et al., 2020a; Shen et al., 2020; Sui et al., 2020a). However, the available lithium resources in the Earth's crust are very limited. By contrast, the reserves of sodium are much more abundant than lithium (Sui et al., 2020b; Zhou et al., 2020). Therefore, the sodium-ion battery is a promising alternative to LIBs, and has attracted extensive attention in recent years (Liu et al., 2021). Nevertheless, the operating voltage and energy density of sodium-ion batteries (SIBs) are generally lower than those of LIBs, as the standard electrode potential of Na/Na^+^ (−2.71 V) is higher than that of Li/Li^+^ (−3.04 V) (Zhu et al., 2013; Wu et al., 2019; Nie et al., 2020b). Obviously, it is very important to develop new cathode materials with high voltages to improve the energy density of SIBs (Wu et al., 2018a; Zheng et al., 2018).

The development of fluorophosphate cathode materials with high working potential and high theoretical capacity has becoming a research hotspot, and various fluorophosphate materials have been reported, including Na_2_MPO_4_F (M = Fe, Mn, Co and Ni) (Barpanda et al., 2018; Li et al., 2018; Wu et al., 2018b), NaVPO_4_F, and Na_3_V_2_(PO_4_)_2_F_3_ (Cai et al., 2018; Subramanyan et al., 2021). Among these fluorophosphate materials, Na_2_MnPO_4_F is attracting significant attention due to its optimal theoretical capacity and suitable working potential. Firstly, the theoretical specific capacity of Na_2_MnPO_4_F is as high as 250 mAh g^−1^ when the two sodium-ions are completely removed (~125 mAh g^−1^ for one sodium-ion) (Wu et al., 2018b; Sharma et al., 2020). Secondly, based on the strong induction effect and strong negative charge of F^−^, Na_2_MnPO_4_F owns high working potential (3.66 and 4.67 V vs. Na/Na^+^) and excellent thermal stability (Wu et al., 2018b), and its working potential is within the stable window of electrolytes. In addition, manganese is low cost and abundant in the Earth. These advantages mean Na_2_MnPO_4_F has a broad application potential. Although Na_2_MnPO_4_F shows such great potential advantages, there are some problems that impede the application of Na_2_MnPO_4_F. Na_2_MnPO_4_F is a broadband insulator, and the low electronic conductivity seriously affects its electrochemical performance. For example, the Na_2_MnPO_4_F sample synthesized by solid state method (Ellis et al., 2010) shows almost no electrochemical activity. At present, there are few reports on the modification of Na_2_MnPO_4_F cathode materials. Most recently, we have prepared a series of solid solution materials of Na_2_Mn_1−x_Fe_*x*_PO_4_F/C (0 < *x* < 1) by introducing Fe element and coating with carbon to improve the electrochemical properties of Na_2_MnPO_4_F (Tang et al., 2020). Our results show that the introduction of Fe and carbon coating can significantly improve the electrochemical activity of Na_2_MnPO_4_F. In a series of the Na_2_Mn_1−x_Fe_*x*_PO_4_F/C samples, Na_2_Fe_0.6_Mn_0.4_PO_4_F/C shows the best electrochemical performance. However, the above report did not investigate the effect of carbon sources on the structure and properties of the material, and did not optimize the type of carbon source. Thus, this paper aims to optimize the carbon source to increase the porosity of Na_2_Fe_0.6_Mn_0.4_PO_4_F/C, optimize the carbon coating effect to inhibit the primary particle overgrowth, and thus build a good conductive network to further improve the electrochemical performance of Na_2_Fe_0.6_Mn_0.4_PO_4_F.

## Experimental

### Chemicals

Sodium carbonate (Na_2_CO_3_, 99.8%) was purchased from Aladdin. Iron citrate (FeC_6_H_5_O_7_, A.R.), manganese acetate tetrahydrate (Mn(CH_3_COO)_2_·4H_2_O, A.R.), ammonium dihydrogen phosphate (NH_4_H_2_PO_4_, A.R.), sodium fluoride (NaF, 99%), oxalate acid (H_2_C_2_O_4_, 99.8%), ascorbic acid (C_6_H_8_O_6_, 99.8%), citric acid (C_6_H_8_O_7_, 99.5%), and glucose (C_6_H_12_O_6_, 99.5%) were purchased from Sinopharm Chemical Reagent Co., Ltd. All chemicals were used directly without further purification.

### Materials Synthesis

The Na_2_Fe_0.6_Mn_0.4_PO_4_F/C composite materials were synthesized by a simple spray-drying method. Firstly, 3.18g Na_2_CO_3_, 3.45 g NH_4_H_2_PO_4_, 1.26 g NaF, 4.41 g C_6_H_5_O_7_Fe·H_2_O, 2.23 g Mn(CH_3_COO)_2_·4H_2_O, and carbon source (1.98 g oxalate acid, 3.87 g ascorbic acid, 4.72 g citric acid, or 4.35 g glucose) were dissolved in 200 ml deionized water to form a mixed solution. Considering the burning loss, the theoretical mass of carbon sources was calculated as 10 wt% of the product (Na_2_Fe_0.6_Mn_0.4_PO_4_F/C). Secondly, the above solution was dried by a spray drier (Shanghai Attainpak DC1500) to prepare the spherical precursor powders. The spraying speed is 250 ml h^−1^, and the inlet and outlet air temperatures are 200° and 80°C, respectively. Finally, the precursor powders were heated at 300°C for 3 h and then calcined at 625°C for 6 h in an argon atmosphere to obtain the final Na_2_Fe_0.6_Mn_0.4_PO_4_F/C products.

### Characterization

The crystalline structure of Na_2_Fe_0.6_Mn_0.4_PO_4_F/C was studied by X-ray diffraction (XRD, Rigaku Ultima IV). The morphology of samples was observed by scanning electron microscope (SEM, Hitachi SU5000). Carbon content of Na_2_Fe_0.6_Mn_0.4_PO_4_F/C was measured with a carbon sulfur analyzer (HCS-140).

### Battery Fabrication and Electrochemical Tests

Firstly, Na_2_Fe_0.6_Mn_0.4_PO_4_F/C powders and acetylene black and poly (vinylidene fluoride) in the weight ratio of 8:1:1 were blended in N-methyl pyrrolidinone to form a homogeneous slurry, then the slurry was spread uniformly on an aluminum foil and dried at 120°C for 12 h in vacuum. The dried foil was pressed into discs (Φ = 12 mm) to act as the cathode plate. An Na-foil (Φ = 14 mm) and glass fiber film disc (Φ = 16 mm) were used as the anode and separator, respectively. A solution of 1 M NaClO_4_ dissolved in 95% PC and 5% FEC was used as the electrolyte. The cells were tested with LAND battery test system at room temperature between the voltage range of 1.5–4.5 V (vs. Na/Na^+^). The electrochemical impedance spectroscopy (EIS, amplitude: 5 mV; frequency: 0.01–100 kHz) was measured by a CHI660D electrochemical workstation.

## Results and Discussion

**Figure 1:**
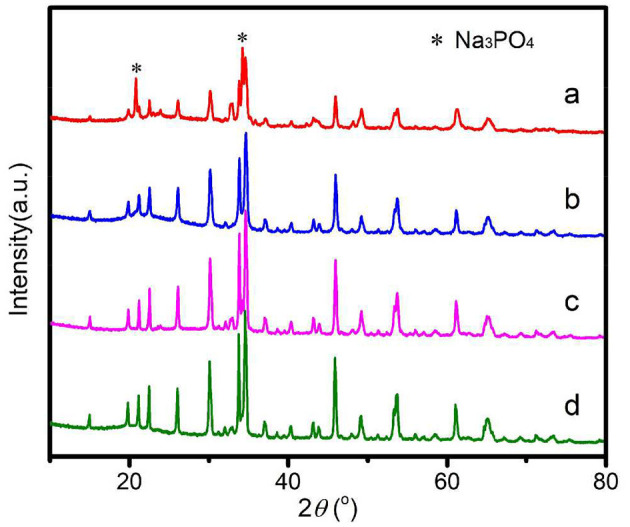
XRD patterns of Na_2_Fe_0.6_Mn_0.4_PO_4_F/C prepared with various carbon sources. **(a)** Oxalic acid; **(b)** Ascorbic acid; **(c)** Citric acid; **(d)** Glucose.

The XRD patterns of Na_2_Fe_0.6_Mn_0.4_PO_4_F/C synthesized with different carbon sources are shown in Figure 1. As shown, all diffraction peaks of the samples can be fully indexed to typical Na_2_Fe_0.6_Mn_0.4_PO_4_F material (PDF#87-0467) with space group *P2*_1_*/n*. The Na_2_Fe_0.6_Mn_0.4_PO_4_F/C samples synthesized with ascorbic acid, citric acid, and glucose were all pure and no impurities were detected. However, for the Na_2_Fe_0.6_Mn_0.4_PO_4_F/C sample prepared with oxalic acid, the diffraction peaks of Na_3_PO_4_ (PDF#71-1918) can be observed, indicating that impurities are produced in the preparation of the Na_2_Fe_0.6_Mn_0.4_PO_4_F/C sample. There are no obvious diffraction peaks related to C that can be observed in the XRD patterns, mainly because the carbon in Na_2_Fe_0.6_Mn_0.4_PO_4_F/C is amorphous. The carbon contents of Na_2_Fe_0.6_Mn_0.4_PO_4_F/C samples prepared with oxalic acid, ascorbic acid, citric acid, and glucose are determined by carbon sulfur analyzing instrument, and the results are 7.1, 7.4, 7.9, and 8.1 wt%, respectively. It can also be seen that the synthesized Na_2_Fe_0.6_Mn_0.4_PO_4_F/C samples with different carbon sources show different peak intensities and widths: the sample prepared with glucose exhibits the highest peak intensity, citric acid takes the second place, and the sample prepared with oxalic acid presents the lowest peak intensity. Normally, high peak intensity and narrow half-peak width indicate large particle sizes of the sample, which will result in unfavorable diffusion and transmission of sodium ions in electrochemical reactions. Interestingly, the order of the reduction ability of the carbon sources in this work is glucose < citric acid < ascorbic acid < oxalic acid, and the difference of reduction ability of four different carbon sources might cause a discrepancy of peak intensity and width. In other words, the relatively high reduction ability of oxalic acid made it difficult to prepare pure Na_2_Fe_0.6_Mn_0.4_PO_4_F/C material.

**Figure 2:**
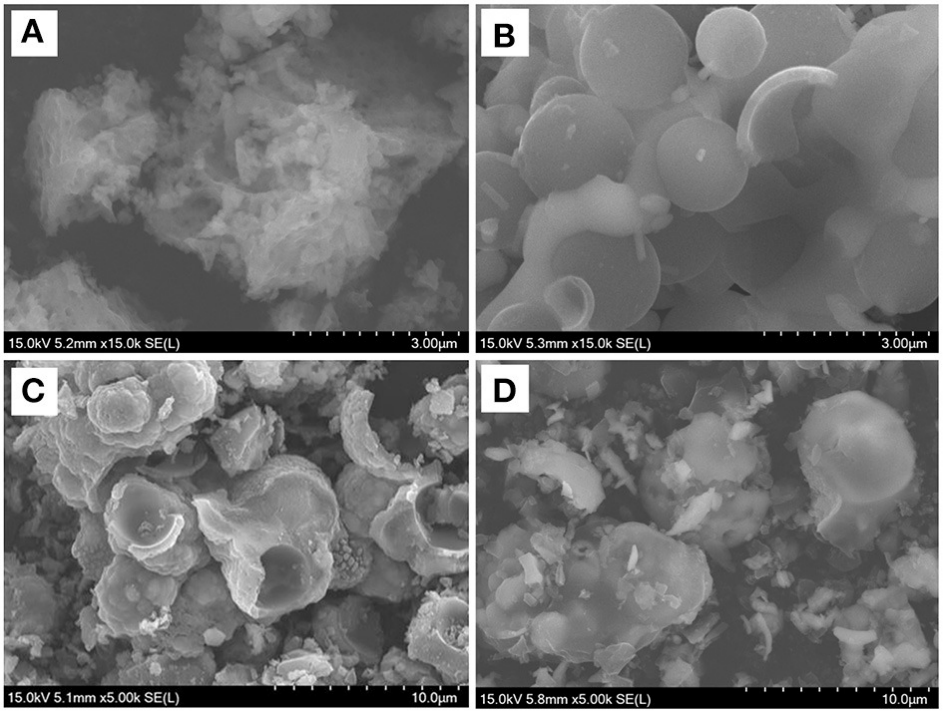
SEM images of Na_2_Fe_0.6_Mn_0.4_PO_4_F/C prepared with various carbon sources. **(A)** Oxalic acid; **(B)** ascorbic acid; **(C)** citric acid; **(D)** glucose.

SEM was employed to analyze the structure and morphology of synthesized Na_2_Fe_0.6_Mn_0.4_PO_4_F/C samples with different carbon sources. It is obvious that carbon sources play a key role in structural construction of Na_2_Fe_0.6_Mn_0.4_PO_4_F/C material. As shown in Figure 2A, the synthesized Na_2_Fe_0.6_Mn_0.4_PO_4_F/C material with oxalic acid exhibits an irregular porous agglomerates morphology with an unclear crystal border, and no spherical structure was observed. It is noted that the formation of the porous structure might be relevant to the decomposition of oxalic acid. The Na_2_Fe_0.6_Mn_0.4_PO_4_F/C material prepared with ascorbic acid, as shown in Figure 2B, displays a hollow spherical shape with a compact surface, and the size distribution is in the range of 1–2 μm. For the Na_2_Fe_0.6_Mn_0.4_PO_4_F/C material prepared with citric acid (Figure 2C), the particles fail to keep the uniform hollow spherical shape, which gradually turned into irregular spheres/agglomerates. Using glucose as a carbon source, the synthesized Na_2_Fe_0.6_Mn_0.4_PO_4_F/C material (Figure 2D) exhibits a damaged spherical shape with irregular particles, and the average size of the damaged spheres is about 3–5 μm. Interestingly, the particle size is decreased with the increase of reduction ability of carbon sources. This phenomenon may provide guidance for the synthesis and optimization of Na_2_Fe_1−x_Mn_*x*_PO_4_F series materials.

**Figure 3:**
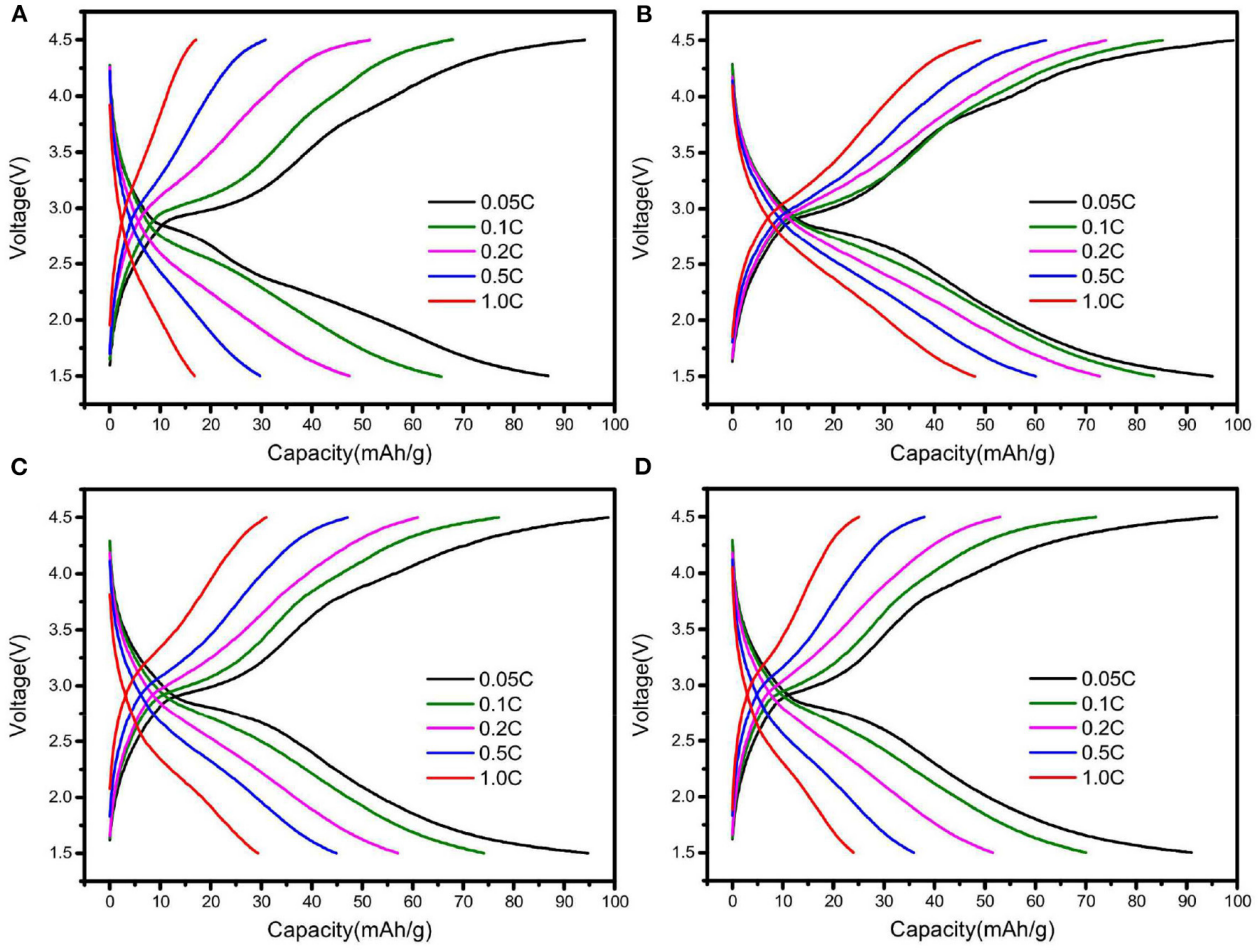
Charge and discharge curves of Na_2_Fe_0.6_Mn_0.4_PO_4_F/C samples prepared with various carbon sources: **(A)** oxalic acid; **(B)** ascorbic acid; **(C)** citric acid; **(D)** glucose.

The rate performance of Na_2_Fe_0.6_Mn_0.4_PO_4_F/C materials synthesized with different carbon sources is shown in Figure 3. As shown in Figure 3A, the Na_2_Fe_0.6_Mn_0.4_PO_4_F/C material prepared with oxalic acid delivers a specific capacity of 86.9 mAh g^−1^ at 0.05C, and the corresponding coulombic efficiency is 92.3%. The discharge capacities can achieve 65.7, 47.5, 29.8, and 16.8 mAh g^−1^ at current densities of 0.1, 0.2, 0.5, and 1C, respectively (Figure 3A). For the Na_2_Fe_0.6_Mn_0.4_PO_4_F/C material prepared with ascorbic acid, the discharge capacities rise to 95.1, 83.5, 72.7, 60.0, and 48.1 mAh g^−1^ at current densities of 0.05, 0.1, 0.2, 0.5, and 1C, respectively (Figure 3B). However, the rate performance of Na_2_Fe_0.6_Mn_0.4_PO_4_F/C material prepared with citric acid and glucose is unsatisfactory (e.g., the discharge capacities are only 30 and 24.0 mAh g^−1^ at 1C, Figures 3C,D). Obviously, the discharge capacities and rate performance of Na_2_Fe_0.6_Mn_0.4_PO_4_F/C materials are closely related to the type of carbon sources, and the material prepared with ascorbic acid shows improved rate performance compared with the materials prepared with the other three carbon sources.

**Figure 4:**
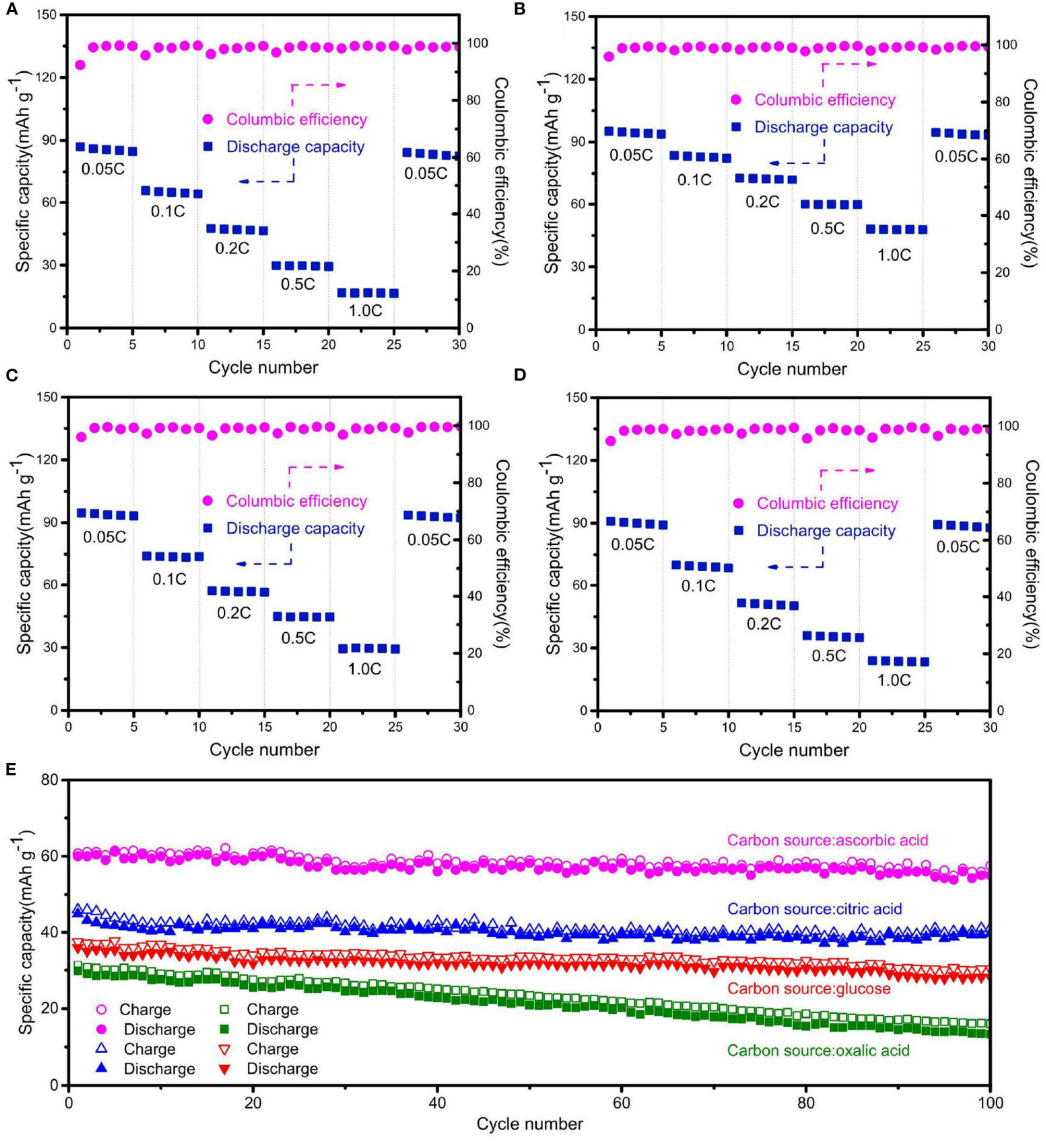
Rate-cycling curves of the Na_2_Fe_0.6_Mn_0.4_PO_4_F/C samples prepared with **(A)** oxalic acid, **(B)** ascorbic acid, **(C)** citric acid, and **(D)** glucose. **(E)** Long-term cycling performance of Na_2_Fe_0.6_Mn_0.4_PO_4_F/C samples at 0.5C rate.

Figures 4A–D shows the rate-cycling performance of Na_2_Fe_0.6_Mn_0.4_PO_4_F/C synthesized with different carbon sources. All Na_2_Fe_0.6_Mn_0.4_PO_4_F/C samples exhibit stable cycle performance at high current densities. Normally, a low current density corresponds to deep charge/discharge behavior, which may cause volume expansion and the irreversible structural change of cathode material. Conversely, a high current density corresponds to shallow charge/discharge behavior, and the structural change of the cathode material is unapparent. The discharge capacity of Na_2_Fe_0.6_Mn_0.4_PO_4_F/C sample prepared with ascorbic acid is as high as 95 mAh g^−1^ after the test, reverting to 0.05C after cycling at various current rates, while the values are only 84, 93, and 88 mAh g^−1^ for the materials prepared with oxalic acid, citric acid, and glucose. The long cycle performance of Na_2_Fe_0.6_Mn_0.4_PO_4_F/C samples synthesized with different carbon sources are shown in Figure 4E. The material prepared with ascorbic acid endures 91.7% of its initial capacity after 100 cycles at 0.5C, while only 44.6%, 88.6%, and 78.3% of the initial capacities are retained for the materials synthesized with oxalic acid, citric acid, and glucose. The result further demonstrates that the Na_2_Fe_0.6_Mn_0.4_PO_4_F/C material prepared with ascorbic acid exhibits outstanding electrochemical performance.

**Figure 5:**
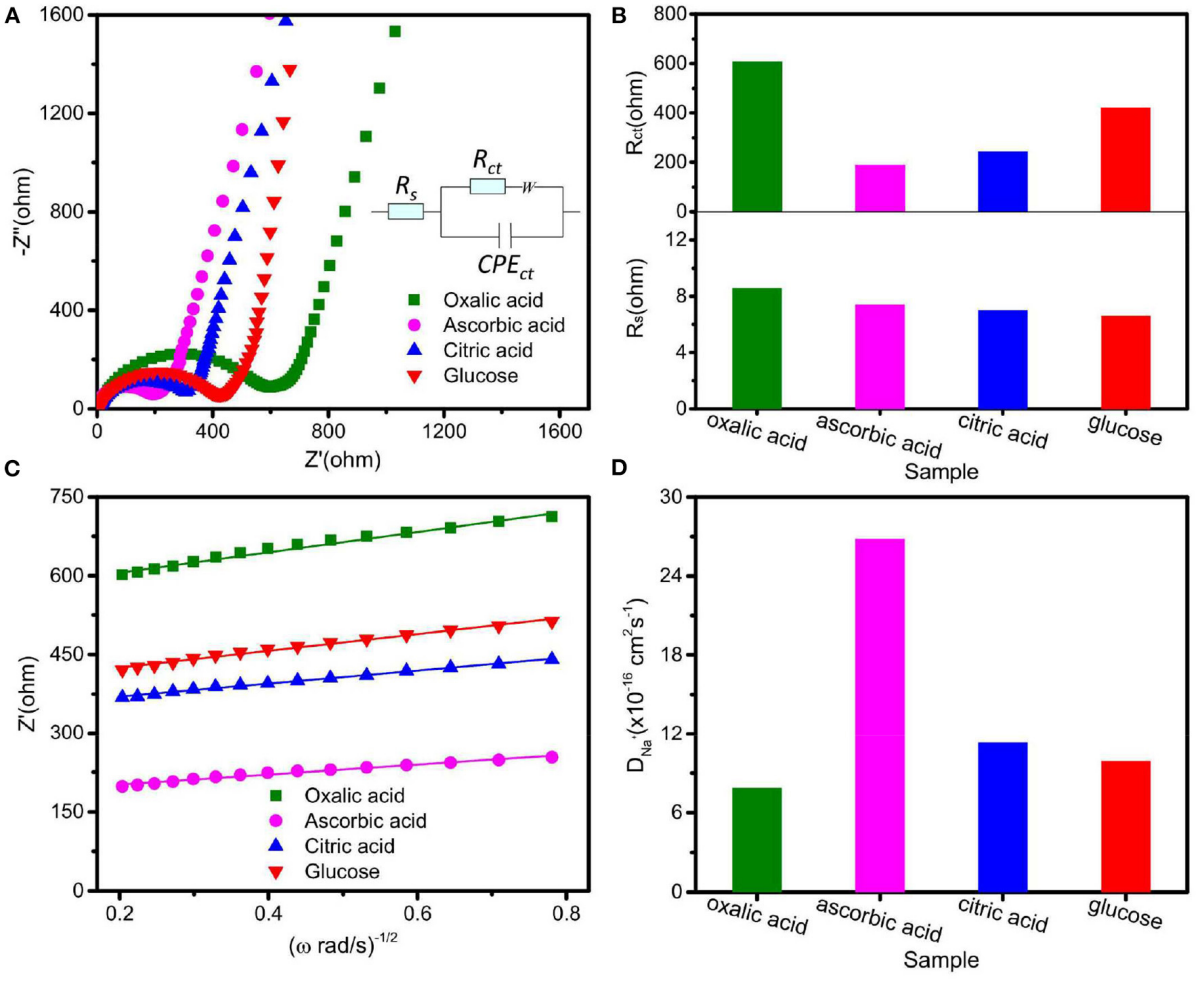
**(A)** Nyquist plots of the prepared electrodes at first full-discharge state with equivalent circuit inset; **(B)** fitting parameters of Nyquist plots; **(C)** relationship between *Z*′ and ω^−1/2^; **(D)** Na^+^ diffusion coefficients of the prepared electrodes.

In order to further reveal the effect of various carbon sources on the electrochemical properties of Na_2_Fe_0.6_Mn_0.4_PO_4_F/C samples, electrode reaction kinetics were investigated by EIS. Figure 5A shows Nyquist plots of four synthesized Na_2_Fe_0.6_Mn_0.4_PO_4_F/C by using an equivalent circuit, and all curves consist of a diagonal line and a semicircle. In the equivalent circuit, *R*_s_ and *R*_ct_ represent the equivalent ohmic resistance of the cell and charge transfer, respectively. Considering the surface of the working electrode is not absolutely smooth, CPE is used to describe the double layer capacitance, and W refers to Warburg impedance (Cui et al., 2016). As can be seen from Figure 5B, the equivalent ohmic resistance (*R*_s_) of these four Na_2_Fe_0.6_Mn_0.4_PO_4_F/C materials is similar, while the material prepared with ascorbic acid exhibits much lower charge-transfer resistance (*R*_ct_) than the other three synthesized materials, which indicates that the material prepared with ascorbic acid presents the lowest electrochemical polarization.

The sodium ion diffusion coefficient (*D*) was also calculated by the following formula.

(1) $$\begin{array}{l} D=R^{2}T^{2}/2A^{2}n^{4}F^{4}C^{2}\sigma_{w}^{2} \end{array}$$

(2) $$\begin{array}{l} Z^{\prime }=R_{s}+R_{ct}+\sigma_{w}\omega^{-1/2} \end{array}$$

Where *R, T, A, n, F, C*, σ_*w*_, and ω represent the gas constant, the absolute temperature, the electrode surface area, the charge transfer number of the electrochemical reaction, Faraday's constant, concentration of Na^+^ ions, Warburg impedance coefficient, and angular frequency, respectively. By calculation (as shown in Figures 5C,D), it is found that the material prepared with ascorbic acid presents the highest sodium ion diffusion coefficient, 2.68 × 10^−15^ cm^2^ s^−1^, which might be due to its high contact area between the electrode material and electrolyte as well as its low crystallite size. The impurities are unfavorable for the sodium ion diffusion; that is why the Na_2_Fe_0.6_Mn_0.4_PO_4_F/C sample prepared with oxalic acid shows the lowest sodium ion diffusion coefficient. These results indicate that the Na_2_Fe_0.6_Mn_0.4_PO_4_F/C prepared with ascorbic acid exhibits high specific capacity, improved rate performance, and excellent cycle stability, and it is expected to be a promising cathode material with high energy density and power density.

## Conclusions

In summary, the effect of various carbon sources on the structure and electrochemical properties of synthesized Na_2_Fe_0.6_Mn_0.4_PO_4_F/C cathode materials is investigated in this study. The results show that the reduction ability of carbon sources plays a key role in the morphology of Na_2_Fe_0.6_Mn_0.4_PO_4_F/C materials, and the material prepared with ascorbic acid presents the most perfect hollow spherical shape. The electrochemical tests demonstrate that the Na_2_Fe_0.6_Mn_0.4_PO_4_F/C material prepared with ascorbic acid exhibits outstanding electrochemical performances. Its high discharge capacities (95.1 mAh g^−1^ at 0.05C), improved rate property (48.1 mAh g^−1^ at 1C), and long cycle stability (endures 91.7% of its initial capacity after 100 cycles at 0.5C) specifically are superior to Na_2_Fe_0.6_Mn_0.4_PO_4_F/C materials prepared with other carbon sources. Electrode reaction kinetics show that the material prepared with ascorbic acid presents lower electrochemical polarization than other materials, which is in accordance with the results of electrochemical tests. This study points out the relationship between the reduction ability of carbon sources and electrochemical properties of Na_2_Fe_0.6_Mn_0.4_PO_4_F/C materials, and it also provides a promising strategy to achieve cathode materials with high energy density and power density.

## Data Availability Statement

The original contributions presented in the study are included in the article/supplementary material, further inquiries can be directed to the corresponding author/s.

## Author Contributions

ST, YS, and LW did the main experiment and wrote the manuscript. YS and LW were involved in the discussion of the experiment and revised the manuscript and also provided the financial support. XZ and BW assisted with the material synthesis and properties analysis. All authors contributed to the article and approved the submitted version.

## Conflict of Interest

The authors declare that the research was conducted in the absence of any commercial or financial relationships that could be construed as a potential conflict of interest.
